# Diffuse Large B-Cell Lymphoma of the Frontal Sinus: A Case Report

**DOI:** 10.3390/hematolrep15030055

**Published:** 2023-09-12

**Authors:** Anastasia Urbanelli, Francesca Testi, Giuseppe Riva, Giancarlo Pecorari

**Affiliations:** Division of Otorhinolaryngology, Department of Surgical Sciences, University of Turin, Via Genova 3, 10126 Turin, Italy; anastasia.urbanelli@unito.it (A.U.); francesca.testi@unito.it (F.T.); giancarlo.pecorari@unito.it (G.P.)

**Keywords:** frontal sinus, lymphoma, diffuse large B-cell lymphoma, paranasal sinus tumor, sinonasal neoplasm

## Abstract

Diffuse large B-cell lymphoma (DLBCL) is the most common type of Non-Hodgkin Lymphoma (NHL). It often involves the gastrointestinal tract, head and neck, and skin, but virtually any tissue or organ can be affected. The primary NHL of the nasal cavity and paranasal sinuses are extremely rare, causing diagnostic and therapeutic difficulties. We present the case of a 49-year-old woman with a 4-week history of diplopia and right superior eyelid swelling. Clinical, radiological, and histological examination led to the diagnosis of DLBCL of the right frontal sinus with anterior invasion of subcutaneous soft tissues and posterior intracranial involvement of the frontal region. She underwent three cycles of MATRIX chemotherapy, three cycles of R-DA-EPOCH, and CAR-T therapy. Unfortunately, treatments were unsuccessful and the patient died 11 months after diagnosis. In conclusion, an early diagnosis of DLBCL of the frontal sinus is difficult as it is often confused with other nasal pathologies. This causes a delay in treatment.

## 1. Introduction

Primary NHLs of the nasal cavities and paranasal sinuses are extremely rare, representing, in the Western world, about 1.5% of all NHL [1,2,3]. Most cases occur in the maxillary sinus, followed by the ethmoid and the nasal cavity. Only 0.17–1.63% of all lymphomas affect the frontal sinus [3]. Diffuse large B-cell lymphoma (DLBCL) represents the most common type of NHL worldwide, accounting for approximately 30–40% of all cases [4]. Furthermore, it is the most common sub-type of lymphoma involving the sinonasal tract [5].

Presenting symptoms are usually non-specific and represented by nasal obstruction, rhinorrhea, epistaxis, acute sinusitis with or without diplopia, exophthalmos, and proptosis. Therefore, sinonasal lymphomas are often confused with more common benign nasal conditions, leading to delayed diagnosis and treatment [6]. In Western countries, paranasal sinus NHLs are mostly represented by the B-cell tumors, while T-cell lymphomas are predominant in Asian countries [7].

In this report, we describe the case of 49-year-old woman with primary DLBCL located in the frontal sinus with right orbital invasion.

## 2. Case Report

**Figure 1:**
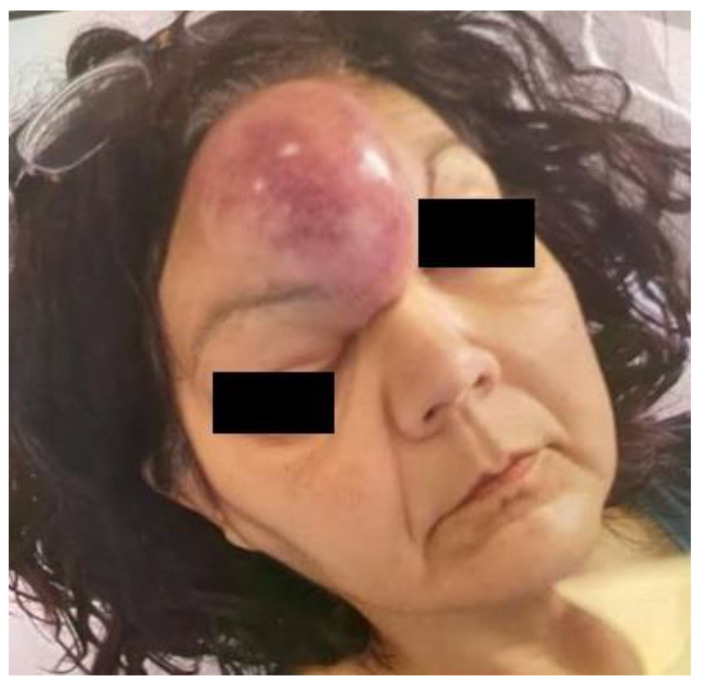
Patient presentation: 5 cm hard fixed bulging in the frontal region determining right proptosis.

In February 2021, a 49-year-old woman presented at our division with a 4-week history of diplopia accompanied by a progressively increasing right frontal bulging that involved the ipsilateral upper eyelid. She had no history of chronic rhinosinusitis and denied nasal symptoms such as nasal obstruction and epistaxis. The visual acuity was normal. No lymphadenopathies were present at clinical examination. The patient did not have any comorbidities and did not complain about B symptoms. She reported discontinuous right ocular pain, partially responsive to acetaminophen. A 5 cm hard fixed bulging in the frontal region was present, which was the cause of the right proptosis (Figure 1). A flexible fiber-optic nasal endoscopy did not show discharge or masses in both nasal cavities.

**Figure 2:**
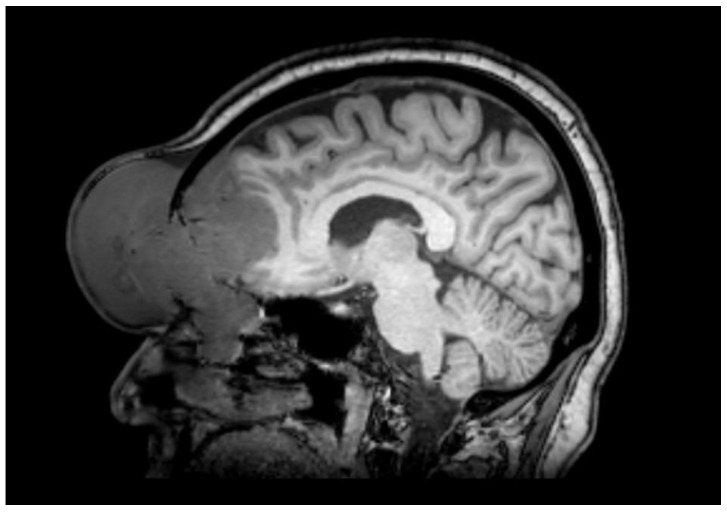
Magnetic resonance imaging (MRI): uniformly enhanced mass in the right frontal sinus with anterior invasion of subcutaneous soft tissues, posterior intracranial involvement, and erosion of the anterior ethmoidal roof and lamina papyracea.

Computed tomography (CT) highlighted a 56 × 36 mm soft tissue mass, characterized by an inhomogeneous contrast enhancement, occupying the right frontal sinus and involving the medial wall of the ipsilateral orbital cavity with erosion of both anterior and posterior wall of the frontal sinus. The mass got close to the frontal encephalic lobe without a clear distinction plane from the adjacent brain structures. CT scans also showed enlarged and partially colliquate lymph nodes in the right neck (levels II-IV sec. Robbins). Magnetic resonance imaging (MRI) confirmed a uniformly enhanced mass in the right frontal sinus with anterior invasion of subcutaneous soft tissues and posterior intracranial involvement (Figure 2). The mass appeared as isointense in T1- and T2-weighted scans. An erosion of the anterior ethmoidal roof and lamina papyracea was observed, with intraorbital invasion and the involvement of superior oblique muscle, superior rectus muscle, and the levator palpebrae superioris muscle. The bulbus oculi was inferiorly and laterally dislocated. No encephalic edema was present.

An endoscopic biopsy under general anesthesia using a neuronavigation system was performed to avoid the risk of a transcutaneous tumor spread present in an open approach. A pathological exam revealed the nodular infiltration of atypical and large-sized lymphocytes with necrosis. The lymphocytes had light basophilic cytoplasm, large irregular nuclei, vescicular chromatin pattern, and multiple nucleoli. Immunohistochemical staining revealed positivity for CD20, MUM1, BCL2, and BCL6 (70% of cells) but not for CD5, CD10, and CD30. The MIB1 labeling index was 80%. The diagnosis was DLBCL of the frontal sinus, non-GCB (Germinal Center B Cell-Like). Fluorescence in situ hybridization (FISH) was negative for translocations of MYC, BCL2, and BCL6 regions. However, duplication of these regions was found in 60% of nuclei.

Positron emission tomography (PET) confirmed the frontal hypermetabolic lesion and the intraparotid metastasis. A bone marrow biopsy did not show any infiltration. The tumor was diagnosed as stage IVE DLBCL according to the Ann Arbor classification, while the International Prognostic Index was 3. Test for human immunodeficiency virus (HIV) was negative.

**Figure 3:**
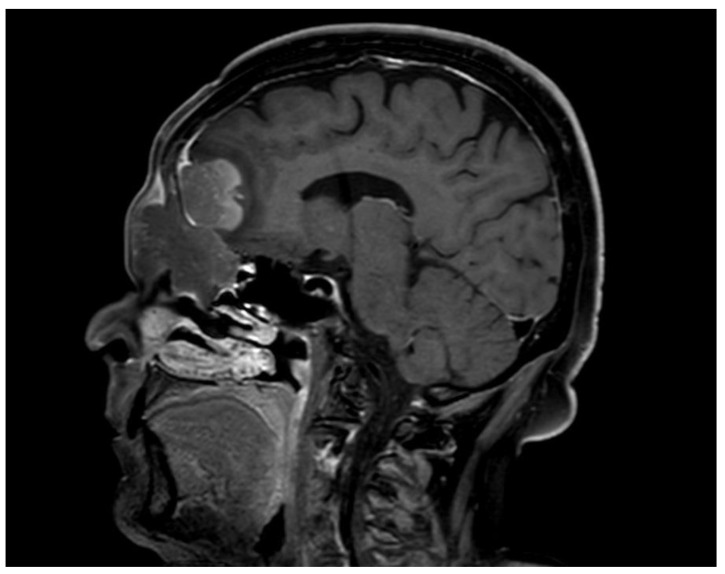
Magnetic resonance imaging (MRI) showing a partial response after chemotherapy with R-DA-EPOCH (etoposide, prednisone, vincristine, cyclophosphamide, doxorubicin, and rituximab).

The chemotherapy protocol MARIETTA was started and included three cycles of the MATRIX regimen (Rituximab-Methotrexate-ARAC-Thiotepa), followed by three cycles of the R-ICE regimen (Rituximab-Isophosphamide-Carboplatin-Etoposide), an autologous stem-cell transplant, and whole-brain radiation therapy. After three cycles of the MATRIX regimen, clinical and radiological progression of disease was observed with right supraorbital extension and retroauricular ipsilateral adenopathy. Therefore, a chemotherapy with R-DA-EPOCH (etoposide, prednisone, vincristine, cyclophosphamide, doxorubicin, and rituximab) was started, with a partial response and decrease in frontal bulging (Figure 3). After two cycles of this regimen, a supraorbital progression was notified.

In August 2021, the patient underwent lymphocyte apheresis for CAR-T (chimeric-antigen-receptor-engineered T cell) therapy (Creative Biolabs, London, UK). In September, radiotherapy and concurrent therapy with pixantrone were performed, with minimal reduction in the supraorbital mass and clinical progression of retroauricular adenopathy. Serologic analysis revealed G2 neutropenia and G1 thrombocytopenia. In October 2021, CART-T cells therapy was performed without objective response. Unfortunately, a massive progression with intracranial invasion occurred in December 2021. Despite all the treatments, the patient passed away in January 2022. Palliative supportive therapy was administered after the massive progression.

## 3. Discussion

NHLs that primarily affect the frontal sinus are rare, as they generally represent the secondary extension of malignancies originating from other sinuses. Only 17 cases have been reported in the literature, and the majority were DLBCL. Frontal sinus NHL may be associated with HIV [8,9].

CT and MRI are necessary to investigate sinonasal diseases. However, a definitive diagnosis can only be achieved with a biopsy. Indeed, a differential diagnosis with other benign and malignant tumors should be taken into consideration. In our case, CD20 positivity was compatible with a B lineage, while it was negative for T-lineage markers like CD5, CD10, and CD30. MRI plays a fundamental role in the evaluation of nasal tumors. Indeed, it helps in the assessment of the multicompartimental extension of the malignancy, and in the correct visualization of its intracranial or perineural spread. For these reasons, MRI is also important in planning adequate treatment for these tumors and in decreasing their intrinsic morbidity, thereby improving the functional outcomes of the proposed therapy. Due to the rapidity of its growth, DLBCL could be confused with a malignant melanoma of the sinonasal tract, which is microscopically composed of pleomorphic spindle cells with hyperchromatic nuclei or of pleomorphic, epithelioid cells and eosinophilic cytoplasm. Other sinonasal malignancies that could be placed in differential diagnosis with DLBCL of the sinonasal tract are neuroendocrine carcinoma, olfactory neuroblastoma (also called esthesioneuroblastoma), Ewing sarcoma, and paraganglioma of the paranasal sinuses. All these disorders share the same aspecific symptomatology that is generally characterized by nasal obstruction, nasal discharge, ocular chemosis or proptosis, postnasal drip, and frequent paranasal infections. These symptoms are also typical of benign conditions such as chronic rhinosinusitis with or without nasal polyps.

In the past, attempts have been made to treat sinonasal lymphomas with radiotherapy, obtaining a short-term clinical response and high incidence of local or distant recurrences [5]. The use of chemotherapy, sometimes associated with immunotherapy, lowered local recurrence, and metastases, improved overall survival [6,10]. Nagafuji et al. described a 67-year-old woman with a left frontal sinus lymphoma diagnosis. She was treated with chemotherapy and immunotherapy based on the R-CHOP protocol (rituximab, cyclophosphamide, doxorubicin, vincristine, prednisone) with no evidence of local or distant recurrence at the 12-month follow-up [10]. Kahn et al. reported a case of a 69-year-old man with a DLBCL lymphoma of the right frontal sinus, for whom a treatment with adriamycin, cytoxan, rituxan, and vincristine was set up with no evidence of radiological disease 3 years later [6].

Generally, the R-CHOP regimen is considered as the best choice of treatment for DLBCLs. However, a consistent group of affected patients (30–40%) are refractory to this protocol or will relapse after the initial response [11]. In recent years, several strategies have been developed to improve the outcome for patients affected by DLBCL who demonstrated a lack of response to conventional therapy.

One of the attempts used to improve the efficacy of the first-line treatment was to adopt more intensive chemotherapy regimens. It was carried out by shortening the interval between the cycles (administered every 2 weeks instead of 3 weeks). However, this attempt did not improve the progression-free survival (PFS) and the overall survival (OS) of the treated patients [12]. In a randomized phase III trial carried out in 2017, a second-generation anti-CD20 monoclonal antibody (Obinutuzumab) was adopted in addition to the CHOP protocol (cyclophosphamide, doxorubicin, vincristine, and prednisone without rituximab) or R-CHOP, but no significant survival advantages were detected [13]. Another attempt was the introduction of oral immunomodulatory drugs like lenalidomide to improve the OS of patients who did not respond to conventional therapy. This regimen revealed conflicting results in terms of improved PFS and OS among different trials [14,15]. Similarly, the addition of the proteasome inhibitor Bortezomib or the oral inhibitor of tyrosine kinasi ibrutinib showed disappointing results in improving all the analyzed endpoints [16,17].

In the case of DLBCL recurrence in patients treated with the R-CHOP regimen, the first choice nowadays is represented by at least two cycles of salvage chemotherapy followed by the combination of high-dose chemotherapy (HDC) and autologous stem-cell transplantation (ASCT) [18]. Generally, the salvage therapy consists of the DHAP (dexamethasone, high dose cytarabine, and cisplatin) regimen. Nevertheless, over the years several trials tried to improve the efficacy of this rescue therapy by modifying the salvage chemotherapy. For example, in the phase III CORAL trial a salvage therapy consisting in rituximab, ifosfamide, etoposide, and carboplatin (R-ICE) was proposed, without changes in PFS and OS if compared to DHAP [19]. Similarly, the NCIC-CTG LY.12 trial demonstrated no differences between the DHAP regimen and the GDP (gemcitabine, dexamethasone, and cisplatin) scheme as salvage chemotherapy in terms of OS [20]. The importance of the salvage chemotherapy to associate to the HDC-ASCT protocol in case of recurrence of DLBCL after primary treatment is to ascribe to its rule in the success of the ASCT. Indeed, in both CORAL and NCIC-CTG LY.12 trials, the portion of patients who did not survive until the ASCT phase showed an early failure in the salvage chemotherapy [19,20].

In our case, the treatment was chosen considering the clinical and immunohistochemical features of the lymphoma. In particular, the following characteristics supported our choice: the presence of an NHL located in proximity to the central nervous system, the absence of previous treatment with high-dose methotrexate-based chemotherapy and/or previous radiotherapy, normal blood tests, the absence of comorbidities, and an age between 18 and 70 years. In the phase II “MARIETTA” clinical trial presented by Ferreri et al. in 2021, the combination of MATRIX and R-ICE was proposed for patients with a diagnosis of lymphoma with extensive central nervous system involvement in subjects who did not undergo other chemotherapy regimens [21]. This regimen showed encouraging results, especially in patients with massive central nervous system involvement at diagnosis, by improving their progression-free survival. Another reason that justified the choice of the MARIETTA protocol as first-line therapy was its acceptable toxicity profile. Adverse effects may be neutropenia, anemia, thrombocytopenia, increased susceptibility to infections, hepatotoxicity, nephrotoxicity, mucositis, gastrointestinal disorders, centrale and peripheral neurotoxicity, cardiotoxicity, and vascular events (deep vein thrombosis, pulmonary thromboembolism, or stroke). Their prevalence varies according to the temporal phase of the regimen. For example, grade 4 neutropenia is reported to occur in up to 37% of cases during the first cycle of MATRIX and in 95% of cases during the autologous stem-cell transplantation [21]. However, the majority of the above-mentioned side effects are very rare with a frequency < 1% for hepatotoxicity, nephrotoxicity, neurotoxicity, cardiotoxicity, and vascular events in all phases of treatment [21]. The presented case showed no adverse effects even after she interrupted the therapy scheme after three cycles of the MATRIX regimen due to the progression of radiological and clinical disease.

The lack of efficacy of the treatments administered may be related to the aggressiveness of the pathology. In particular, the progression-free survival and overall survival in patients treated with the MARIETTA protocol for NHL is strictly dependent on a good response to the MATRIX (represented by the first three cycles of the whole treatment), whereas patients with MATRIX-refractory disease (like our presented case) showed little benefit to the whole therapy scheme [21]. Further studies are necessary to better understand the predictive role of clinical and pathological characteristics.

Regarding CAR-T therapy, it consists of an individualized technology that modifies the patients’ T lymphocytes through a CD-19 antigen-specific domain to eradicate malignant cells. This treatment could be used in patients affected by DLBCL who are non-responsive to conventional chemotherapy schemes or patients considered ineligible for hematopoietic stem-cell transplantation [22]. An important prerogative in the adoption of CAR-T therapy is the accurate identification of molecular biomarkers expressed by the tumor (e.g., CD19, CD3, PD-1, PD-L1, CD3, TIM3, and LAG3). Such features allow for a personalized target for every patient treated with this protocol. The side effects associated with the adoption of CAR-T therapy are represented via the Cytokine Release Syndrome (CRS) which is characterized by a generalized inflammatory state triggered by cytokines and chemokines, and the neurotoxicity due to endothelial activation and the disruption of the blood–brain barrier with the consequent entry of inflammatory cytokines into the central nervous system [22].

Besides CAR-T therapy, other new treatment choices are available as salvage therapy in case of the recurrence of DLBCL or in patients who are non-responsive to conventional schemes. For example, the combination of Tafasitamab (anti-CD19 monoclonal antibody) and lenalidomide was tested in patients considered as non-eligible for ASCT. It showed better outcomes in terms of OS in respect to other salvage schemes such as vedotin plus rituximab and bendamustine (Pla-BR) with a reasonable toxicity profile [23]. On the other hand, the Pla-BR regimen showed better efficacy if compared to rituximab and bendamustine alone, with a low rate of adverse events and a safe neurotoxicity profile [24].

In the era of the precision medicine based on the adoption of individualized approaches, tailored therapeutic choices might play a role in the treatment of DLBCL. Generally, three different subtypes of DLBCL have been defined: ABC (activated B cell-like), GCB (Germinal Center B Cell-Like), and unclassified subtypes. More recently, DLBCL was divided into two “molecular classification” with respect to targetable gene alterations, thereby paving the way for a new-era precision medicine [11]. For example, Wilson et al. conducted the PHOENIX trial to identify, among non-GCB DLBCLs, molecular subtypes responsive to the ibrutinib plus R-CHOP regimen. They detected in particular three gene mutations responsible for a better prognosis for patients treated with this scheme: MYD88, CD79b, and NOTCH1 mutations [25]. Another phase II trial divided a group of diagnosed DLBCL into six subgroups (MCD-like, BN2-like, N1-like, EZB-like, TP53-mutated, and others subgroups) on the basis of the different molecular pattern of each group: MCD-like and BN2-like subgroups received ibrutinib, N1-like lenalidomide, EZB-like tucidinostat (a histone deacetylase inhibitor), TP53-mutated decitabine, and the other subgroups received lenalidomide [26]. This new therapeutic approach allows us to build a more appropriate treatment scheme for each molecular DLBCL profile, thereby developing a more efficient target therapy to treat this malignancy.

In conclusion, because of the rarity of the localization, an early diagnosis of DLBCL of the frontal sinus is difficult as it is often confused with other nasal pathologies. This causes a delay in diagnosis and treatment. While the diagnostic assessment is well defined in the literature, the treatment protocols vary. Further investigations are necessary to reach more accurate conclusions regarding the best treatment for these patients.

## Data Availability

Not applicable.
